# Mesenchymal Stromal Cell-Based Products: Challenges and Clinical Therapeutic Options

**DOI:** 10.3390/ijms25116063

**Published:** 2024-05-31

**Authors:** Debora B. Mello, Fernanda Cristina Paccola Mesquita, Danúbia Silva dos Santos, Karina Dutra Asensi, Marlon Lemos Dias, Antonio Carlos Campos de Carvalho, Regina Coeli dos Santos Goldenberg, Tais Hanae Kasai-Brunswick

**Affiliations:** 1 National Center of Structural Biology and Bioimaging, CENABIO, Federal University of Rio de Janeiro, Rio de Janeiro 21941-902, Brazil; debmello@cenabio.ufrj.br (D.B.M.); acarlos@biof.ufrj.br (A.C.C.d.C.); 2 Regenerative Medicine Research Department, The Texas Heart Institute, Houston, TX 77030, USA; fmesquita@texasheart.org; 3 Center of Cellular Technology, National Institute of Cardiology, INC, Rio de Janeiro 22240-002, Brazil; danubia_ss@yahoo.com.br; 4 National Institute of Science and Technology for Regenerative Medicine-REGENERA, Federal University of Rio de Janeiro, Rio de Janeiro 21941-902, Brazil; karina_asensi@biof.ufrj.br (K.D.A.); rcoeli@biof.ufrj.br (R.C.d.S.G.); 5 Carlos Chagas Filho Institute of Biophysics, Federal University of Rio de Janeiro, Rio de Janeiro 21941-902, Brazil; marlonlemos@biof.ufrj.br

**Keywords:** mesenchymal stromal cell, advanced therapy medicinal products, regenerative medicine

## Abstract

Mesenchymal stromal cell (MSC)-based advanced therapy medicinal products (ATMPs) are being tried in a vast range of clinical applications. These cells can be isolated from different donor tissues by using several methods, or they can even be derived from induced pluripotent stem cells or embryonic stem cells. However, ATMP heterogeneity may impact product identity and potency, and, consequently, clinical trial outcomes. In this review, we discuss these topics and the need to establish minimal criteria regarding the manufacturing of MSCs so that these innovative therapeutics may be better positioned to contribute to the advancement of regenerative medicine.

## 1. Introduction

Advanced therapy medicinal products (ATMPs) are innovative therapeutic products for human use that are classified as somatic–cellular medicine, tissue-engineered cell medicine, and gene therapy medicine. ATMPs are extensively manipulated products aimed at regenerating, repairing, or replacing a cell or tissue or even at preventing the development of diseases by the use of their biological, physiological or structural properties [1]. Mesenchymal stromal cells (MSCs) have been widely studied and have emerged as somatic–cellular medicine. The implementation of medical therapies using MSC is fast becoming a reality, but the necessary tight controls on the production, storage, and transport of these cells and the strict criteria for classification of these biological materials as ATMPs need to keep pace. To date, regulatory agencies and scientists have been working together to establish guidelines to mitigate the variability of cell-based products and establish a well-defined regulatory framework for the marketing authorization of ATMPs. This review focuses on the importance of quality control and the establishment of standards in these processes.

Mesenchymal stromal cells, while sharing the same acronym as mesenchymal stem cells, multipotential stromal cells, mesenchymal progenitor cells, and medicinal signaling cells, do not necessarily represent the same cell population. Here, we use the definition recommended by the International Society for Cell & Gene Therapy (ISCT): mesenchymal stromal cells (hereafter “MSCs”) are a heterogeneous cell population of mesenchymal origin, residing in every organ of the body containing a perivascular niche, including fibroblasts, myofibroblasts, and a small portion of stem/progenitor cells [2]. MSCs have differentiation capabilities in three lineages (osteoblasts, adipocytes, and chondrocytes), as well as a homing ability. Importantly, MSCs can modulate the innate and adaptative immune systems via paracrine factors and direct cell–cell contact; they secrete bioactive factors, functioning as a “cytokine pump” by releasing microvesicles/exosomes (containing biologically active proteins, mRNAs, and miRNAs) and transferring mitochondria [3,4,5]. These features of MSCs act together to modulate the injured tissue microenvironment.

MSC-based therapies in a vast number of disease models have produced beneficial results, serving as the basis of an explosion of clinical studies to evaluate MSC safety, feasibility, and efficacy [6]. The first clinical trial conducted using autologous and allogeneic bone marrow-derived MSCs (BM-MSCs) was performed by Lazarus and colleagues in 1995 to treat patients with hematologic disorders. The phase I study demonstrated the safety and feasibility of using MSCs clinically [7]. These data propelled a massive investment of USD 19.9 billion in 2020 into the regenerative medicine field—a USD 6.4 billion increase over the previous record, which was USD 13.5 billion in 2018. Also, capital investment in the clinical investigations of MSCs reached USD 22.7 billion in 2021 [8]. In addition, scientific interest and the therapeutic promise of MSCs have been demonstrated by the nearly 70,000 publications registered on the subject in the last 10 years.

**Figure 1:**
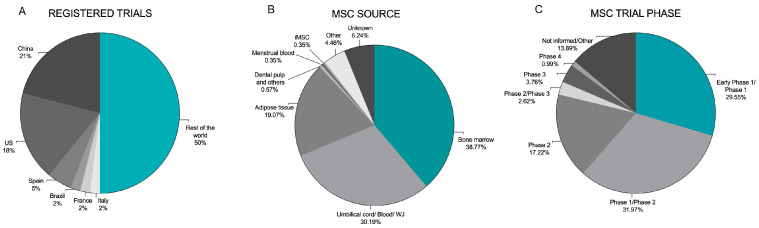
The worldwide distribution of MSC-based therapy. (**A**) Proportion of the clinical trials of MSCs by location. Data were obtained by a search of “mesenchymal cells” in https://clinicaltrials.gov/, (accessed on 22 September 2023). (**B**) Clinical trials were classified according to the trials phase. (**C**) Clinical trials were classified according to the source of MSCs.

Currently, there are 1411 registrations using “mesenchymal cells” as a key word (including stromal and progenitor variations) on the clinicaltrials.gov website. Using this same key word, the International Clinical Trials Registry Platform of the World Health Organization (https://ictrptest.azurewebsites.net/Default.aspx, accessed on 21 September 2023) displayed 1748 registrations. The distribution of those trials worldwide is presented in Figure 1A.

In addition to the diversity of countries involved in trials, there have been variations in the cell sources among the trials, with the MSCs derived from bone marrow (BM), adipose tissue (AT), and umbilical cord (UC), being the most studied (Figure 1B) [9]. Since the publication of differentiation protocols for deriving MSCs from pluripotent stem cells (embryonic and induced) in 2016, these sources of MSCs are gaining interest [10].

The majority of MSC clinical trials performed or currently in progress are phase I or II (Figure 1C) [11]. The success observed in experimental or preclinical studies is not always repeated in clinical trials, so suboptimal outcomes are frequently found in the latter. In cardiovascular clinical trials, for example, the impressive improvement in ejection fractions in MSC-treated acute myocardial infarction in the experimental scenario was not consistently achieved in clinical settings [12,13]. Even in bone and cartilage diseases, where the beneficial outcomes of BM–MSC therapy are more readily seen, controversial results in clinical trials have been observed [14,15]. Since the publication of the results of these initial trials, researchers in the field have pointed out that many of the variables involved in MSC-based therapies, such as donors, intrinsic cell culture characteristics (reagents, plate density, viability, and the establishment of a master cell bank), storage, and transport conditions, can affect the comparisons of the outcomes [16]. It is imperative to produce MSCs as a standardized product and to have their identity, potency, viability, and stability attested to and validated by analytical methods to achieve consistent and reproducible results in the clinical setting.

The workflow management for MSC-based products considers the cells to be an innovative class of medicine for human use that encompasses gene therapy, bioengineering products, and manipulated somatic–cellular therapy. The US Food and Drug Administration (FDA) was the first to establish rules for the marketing of ATMPs by publishing guidelines for industries and sponsors in 1997 [17]. This milestone legitimized the market through laws implemented in a sequence of countries and their regulatory agencies, such as in Europe (EMA), Japan (PMDA), Brazil (ANVISA), South Korea (MFDS), Argentina (INCUCAI), Australia (TGA), the United Kingdom (MHRA), etc. However, some countries have no relevant legislation established (or no evaluation framework), thus making the process of standardizing these cell-based products even more challenging.

**Figure 2:**
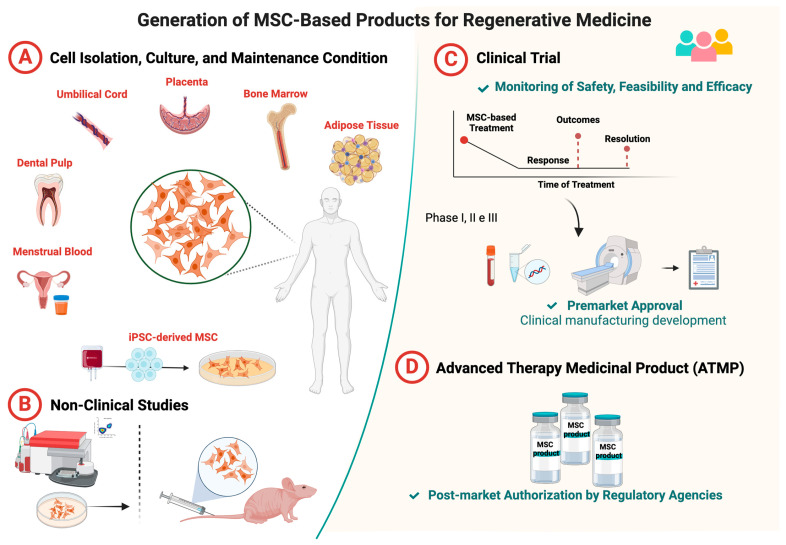
Cell-based ATMP workflow. (**A**) Basic research is the first step to establish an MSC product as an ATMP. In laboratories, the cells are isolated from different sources and extensively studied, and the aim is to understand their properties and potential clinical applications. (**B**) Preclinical safety studies are designed using experimental models of human diseases. (**C**) Once successful in experimental models, MSCs can be tested in humans through clinical trials, which involves phases I, II, and III for safety, feasibility, and efficacy evaluation. An MSC can only be defined as an ATMP after evaluation by a regulatory agency and approval for commercialization. (**D**) The ATMP commercialization process is also assessed by the regulatory agency through post-market evaluation.

The life cycle of ATMPs involves research and development stages (Figure 2A), preclinical safety studies (Figure 2B), and clinical safety and effectiveness tests in humans (Figure 2C) until the product is registered as being ready for commercialization (including post-market evaluation) (Figure 2D). There are no unified guidelines for MSC development for clinical applications, but any cell-based product needs to follow manufacturing instructions in accordance with the principles of the Code of Good Manufacturing Practice (cGMP) and the policies and procedures of the internal Quality Management System (QMS), which is based on the requirements of the International Organization for Standardization (ISO). To achieve this goal, a consensus must be reached on common regulatory processes between countries.

Currently, there are regulatory differences among countries, beginning with the nomenclature of cell-based products, e.g., ATMP in the European Union; Human Cells, Tissues, and Cellular and Tissue-Based Products (HCT/P’s) in the USA; Regenerative Medicinal Product in Japan; Biological Drug in Canada; and Cell Therapy Product in Korea. More importantly, discrepancies also exist in defining the criteria of quality and potency [18,19]. Even with more than 1400 MSC studies registered on clinicaltrials.gov and the efforts of agencies around the world to regulate these ATMPs, only 11 products have been licensed for human use. Asia occupies a leading position, being responsible for 8 of them. In this region, the Republic of Korea has 4 licensed MSC products, which is followed by Japan with two products, and India and Iran with one each. They are intended to treat subcutaneous tissue defects [20], Crohn’s fistula [20], myocardial infarction [21], osteoarthritis [22,23], amyotrophic lateral sclerosis [24], graft-versus-host disease (GVHD) [25], spinal cord injuries [26], and critical limb ischemia [27]. Europe has currently two licensed MSC products to treat GVHD [28] and Crohn’s fistula [29], and the USA has only one, for pediatric patients with GVHD [30], as summarized in Table 1. This plethora of applications reflects one of the difficulties that regulatory agencies have to deal with. The legacy-driven requirements for regulatory agencies were based on pharmaceutical drugs and could not be completely applied to cell-based products. As demonstrated above, the same product could be used to treat completely different diseases. Because of that, they should be evaluated differently and based on this action mechanism. Regulatory agencies are frequently publishing specific guidelines, and they also discuss with the scientific community on how to build the basis for evaluating ATMPs [31,32]. One example of such a fruitful partnership can be found in South America, where the Brazilian Health Regulatory Agency (ANVISA) has been advised by a national network of specialists in advanced therapies—members of the scientific community—to support the licensing of ATMPs [33].

In this review, we first discuss the use of MSCs derived from BM, AT, UC and Placenta—the types that have been predominantly used in clinical trials in the past 10 years—in the context of their use as ATMPs. We also discuss the new emerging sources of MSCs, such as menstrual blood, dental pulp, and MSCs derived from induced pluripotent stem cells. We conclude with a discussion of the steps required to obtain a unique MSC product, including the establishment of MSC identity, the manufacturing process, and potency assays, as well as discuss the ongoing research necessary for improving the safety and efficacy of MSC-based therapies, like the route of administration.

## 2. Cell Source Overview

### 2.1. Bone Marrow: The Beginning

The observations made by Friedenstein more than four decades ago concerning the presence of a non-hematopoietic population of cells in postnatal bone marrow opened a new avenue for regenerative medicine. These cells were adherent to plastic, had a fibroblast-like morphology, had a clonogenic ability to form colony-forming unit–fibroblasts (CFU-F), and they presented a multipotent capability to differentiate into adipocytes, chondrocytes, and osteoblasts. Furthermore, in vivo, they provided a hematopoietic supporting stroma even with just a single transplanted CFU-F [35,36]. Due to these special characteristics, in 1991, Caplan named these cells mesenchymal stem cells [37]. In brief, bone marrow MSCs (BM-MSCs) are usually isolated from iliac crest bone of the pelvis through an invasive method requiring anesthesia and the placing of the patient under infection risk. After flushing the bone marrow, the obtained pool of cells is submitted to a density gradient to recover mononuclear cells, which are then plated at low density for cell expansion [38].

Large numbers of preclinical studies using BM-MSCs were initially intended to explore the capability of “stem cells” to treat various diseases, such as diabetes and osteogenesis imperfecta [39,40], ischemic diseases (e.g., myocardial infarction and stroke) [41,42], immune disorders (e.g., GvHD) [43], and infectious diseases (e.g., sepsis and Chagas disease) [44,45]. These studies showed that BM-MSCs comprise an extremely rare stem cell population (0.01 to 0.001% of total bone marrow cells), are able to home to sites of injury or disease, and secrete bioactive molecules that exert immunomodulatory and paracrine effects [46]. Most of the beneficial effects observed in these preclinical studies were not attributed to the regenerative capability of the MSCs to differentiate into cells of the damaged tissues (i.e., the stem cell properties themselves), but rather to the trophic and paracrine effects that they promoted on the microenvironment where they were implanted [46]. Thus, the nomenclature mesenchymal “stem cell” was beginning to be challenged. Furthermore, cells with the same morphological and functional characteristics began to be isolated from other tissues of stromal origin, leading to a new meaning of the acronym MSC (i.e., mesenchymal *stromal* cell); then, “stromal cells” began to be used instead [2].

The potential benefits promoted by the administration of BM-MSCs to treat different disease models quickly pushed their use and evaluation into clinical settings and, with this, the necessity to establish an identity for MSCs. In this regard, Dominici and colleagues published, in 2006, the minimum criteria to define the human BM-MSC on behalf of the ISCT. These criteria are as follows: (1) BM-MSCs must adhere to plastic culture dishes; (2) the cells must express, by flow cytometry, the surface markers CD90, CD73, and CD105 on more than 95% of the cells, and they must also express CD45, CD34, CD14, CD11β, CD79α, CD19, or HLA-DR on less than 2% of the cells; and (3) the cells must have the ability to differentiate into three specific mesodermal cell lineages in vitro (osteoblasts, adipocytes, and chondrocytes) [47].

This ISCT position paper was the first milestone toward moving MSCs into the ATMP category, and it has also paved the way to identify a human BM-MSC. MSCs obtained from other sources are also identified based on these criteria, although slight differences remain regarding surface molecule expression, proteomics, and even the differentiation capabilities of the cells [48,49,50,51,52].

Due to the vast documented knowledge on BM-MSC physiology and the establishment of minimal criteria for BM-MSC definition, this cell source has been widely used in clinical trials worldwide, and it is currently the cell type that is most commonly registered as an ATMP. One of the first BM-MSC ATMPs was registered in Canada in 2012, known as Prochymal^®^ by Osiris Pharmaceuticals (“Remestemcel-L”, later called “Ryoncil” by Mesoblast Ltd.). The product was intended to treat acute GvHD in children as an off-the-shelf stem cell product. Additional investigations were requested in the Biologics License Application (BLA), and a phase III study was conducted by the company [53]. A post hoc analysis of the study suggested few clinically meaningful observations [54]. In October 2020, Ryoncil’s BLA received a complete response letter from the FDA recommending a randomized, controlled study, even though nine out of the ten FDA reviewers had recommended product approval by the Oncologic Drugs Advisory Committee (ODAC) [55]. Recently, in August 2023, the FDA issued a new position requesting more data to support marketing approval [56]. As noted, the path for an MSC to be marketed as an ATMP is not trivial. We summarize the MSC-based therapies currently classified as ATMPs in Table 1.

### 2.2. Placenta and Umbilical Cord

The placenta and umbilical cord (UC) are “extraembryonic attachments” that have been explored for over 20 years as a source of MSCs due to their early embryological origin and easy access for cell procurement since they are discarded postpartum. Human placental cell is a generic term used to refer to any cell type that can be isolated from the full-term placenta, and it includes epithelial cells, MSCs, endothelial cells, and hematopoietic cells [50]. The UC is the connection between the placenta and the fetus, and it consists of two umbilical arteries and one umbilical vein surrounded by mucoid connective tissue. MSCs have been described surrounding the vessels and in the gelatinous substance called Wharton’s Jelly (WJ), one of the most readily accessible UC-MSC sources [57,58,59]. All cited neonatal tissue-derived MSCs have a varying range of maturation (from a mesenchymal to a myofibroblast phenotype [60]), possess enormous proliferative rates [61], and share MSC properties [62]. Protocols to isolate placental (P-MSCs) and UC-MSCs vary according to the site of origin (WJ, UC vein, and placenta) [60], but most methods use enzymatic tissue digestion, although some differences exist between investigators [63,64].

Several preclinical studies have successfully demonstrated beneficial results using P-MSCs. These cells promote angiogenesis and cell proliferation, inhibit apoptosis, attenuate inflammation, activate several signaling pathways through paracrine effects, and improve organ function in various animal models, such as brain injury [65], lung [66], cardiovascular [67,68], and liver disease [69] models, among others [70,71,72].

The regenerative medicine field has used cord blood extraembryonic cells since 2005, and clinical trials with other extraembryonic tissue-derived MSCs have been ongoing since 2009 [73]. Over 450 clinical trials have been registered using placental and/or UC-MSCs in the past 15 years [73,74]. Most of these studies are in phase I and II, with some in phase III and IV. These trials have predominantly been conducted in China, the USA, and South Korea [73,75], and they have been investigating the application of UC-MSC-based therapies in wound healing (NCT04104451 and NCT01221428), inflammatory disorders (NCT02445547 and NCT03059355), immune disorders (NCT03158896 and NCT04061746), neurological disorders (NCT03684122 and NCT04089579), metabolic disorders (NCT02302599 and NCT05003908) and genetic disorders (NCT02235844 and NCT02285673) [61,74]. There have also been UC-MSC-based therapies evaluated for the treatment of cardiovascular (NCT01739777 and NCT01219452), hematopoietic (NCT03899298 and NCT05672420), hepatic (NCT04357600 and NCT02812121), urologic (NCT03899298 and NCT04972890), and respiratory (NCT04288102 and NCT04433104) diseases. Follow-up results for some of these clinical trials are encouraging, particularly for the treatment of autoimmune and endocrine diseases [74,76].

The publication of guidelines for the use of human P-MSCs and UC-MSCs has allowed for the establishment of UC tissue biobanks, making the MSCs from these sources attractive to the industry. The guidelines state that human P-MSCs and UC-MSCs can be isolated, expanded, stored, and released for clinical use if cultivated for a minimum of 14 days in sterile conditions, presenting more than 70% viability, having a normal karyotype, expressing MSC markers (>90% CD73+, >90% CD105+, and <1% CD45+), being Gram stain and mycoplasma negative, having endotoxin levels of < 2 EU/mL, and the donor serology for infectious disease being negative 180 days after procurement [77]. MSCs from placenta and UC are already classified as ATMPs in several countries [75]. The manufacturing of placental- and UC-based ATMPs requires the donation of the tissue and corresponds to the starting material of the ATMP. Cord tissue biobanks must be accredited or authorized by the competent authority of each regulatory agency to procure the tissue [75].

Currently, there are few registered UC- and placenta-related products/trademarks. Cartistem^®^ is a South Korean MFDS-registered UC blood-MSC ATMP that has been indicated for cartilage degeneration. EpiCord^®^ is a commercially available product showing promising results in treating non-healing diabetic foot ulcers [78], but UCX^®^-ATMP, although already trademarked [79], has not yet had its clinical applications disclosed.

### 2.3. Adipose Tissue

Adipose tissue MSCs (AT-MSCs) were first described by Young and colleagues in 1960 [48]. The authors described the following two large cell groups with distinct characteristics that arise following the enzymatic digestion of fat: (1) adipocytes residing in the portion that floats at the end of the collagenase digestion protocol, and (2) another pool of cells found sedimented in the tube after centrifugation (later called the stromal vascular fraction (SVF)). In 2001, Zuk and colleagues demonstrated that SVFs are composed of a collection of different cell types such as endothelial progenitors, preadipocytes, fibroblasts, resident blood cells (macrophages and monocytes), lymphocytes, hematopoietic stem cells, and the mesenchymal cells derived from adipose tissue (which share MSC properties) [80].

A great technical advantage in obtaining AT-MSCs is the routine and low-risk clinical practice of liposuction or lipectomy in plastic surgery as these samples are usually discarded, making these cells an attractive clinical product. Standard protocols use enzymatic digestion with collagenase and red blood cell removal with specific lysis solutions and filtration. After that, the cells are expanded in a similar way to BM-MSCs. Relevant concerns regarding variability include the wide variety of characteristics associated with donors, such as age, comorbidities, genetics, the level of stress to which the cells are subjected, and the anatomical site for fat extraction [81,82,83]. Also, technical aspects such as isolation protocols, plastic adherence properties, time in culture, and enzymatic procedures can modify surface and adhesion molecules, as well as the extracellular matrix proteins and cytoskeleton proteins of AT-MSCs [84,85,86], thus resulting in consequential inter-product variation.

Based on the extensive use of AT-MSCs and on the promising data originating from experimental and clinical trials, they are the third most used MSC in clinical trials worldwide, with the first being BM-MSC and the second being UC-MSCs, as shown in Figure 1C [9]. The use of AT-MSCs in the clinic began in 2002 with Rigotti and colleagues, who successfully used AT-MSCs obtained from the liposuction in patients with severe sequelae due to radiotherapy [87]. A recent search on the clinicaltrials.gov website found 424 trials under the keywords “adipose mesenchymal OR adipose stromal” and “interventional” filter. Those studies propose the autologous and allogeneic use of AT-MSCs in diseases such as limb ischemia (NCT04466007), GvHD (NCT01222039), ischemic stroke (NCT04280003), urinary incontinence (NCT04446884), amyotrophic lateral sclerosis (NCT02492516), aqueous deficient dry eye disease (NCT03878628), liver cirrhosis (NCT02705742), etc. Of those, 149 are already completed, and the rest are ongoing and/or recruiting. The vast majority of these are in early phase I and II (*n* = 300), although more advanced phase trials, III and IV, have been registered, albeit at a much lower number (*n* = 18) and for only three target diseases—perianal fistula (NCT03279081 and NCT04612465), osteoarthritis (NCT03818737, NCT04230902, NCT04351932, NCT04368806, NCT04427930, and NCT04675359) and ischemic heart disease (NCT04005989).

Very few adipose tissue-based ATMPs are commercially available. The first one was Cupistem^®^ in Korea in 2012 [11]. In 2018, the EMA gave market authorization for Alofisel, and there was also a renewal in January 2023 [88]. These products are intended for the recovery of complex perianal fistulas, the lesions in subcutaneous tissue caused by Crohn’s disease. Additionally, six products are being tested in ongoing clinical trials aimed at treating different conditions such as acute traumatic spinal cord injury, acute respiratory distress syndrome, knee osteoarthritis, chronic venous stasis ulcer, Alzheimer’s disease, traumatic brain injury, hypoxic–ischemic encephalopathy, COVID-19, and rheumatoid arthritis [11].

### 2.4. Dental Pulp

The existence of dental MSCs was suggested by the formation of reparative dentin to protect pulp tissue after external damaging stimuli, such as caries and trauma [51]. Approximately eight unique MSC-like populations have been identified from teeth and related supporting tissues [51]. Gronthos and colleagues initially isolated dental pulp mesenchymal stem cells (DP-MSCs) from a human impacted third molar [89]. MSC-like cells have also been isolated and characterized from human exfoliated deciduous teeth (SHEDs) [90], periodontal ligament stem cells (PDLSCs) [91], dental follicle precursor cells (DFPCs) [92], alveolar bone-derived mesenchymal stem cells (ABMSCs) [93], stem cells from apical papilla (SCAP) [94], tooth germ progenitor cells (TGPCs) [95], and gingival-derived mesenchymal stem cells [96].

In comparison to other dental MSC-like populations, DP-MSCs and SHEDs are more attractive as cell sources for clinical use due to their easy accessibility from extracted teeth, which are considered disposable during orthodontic treatment [97,98]. Also, the isolation methods of DP-MSCs and SHEDs use enzymatic digestion, which are also used to isolate the MSCs derived from adipose tissue [86,87].

Due to their excellent potential for multilineage differentiation, DP-MSCs and SHEDs have been used to treat dental diseases in several experimental studies [99,100,101,102,103], demonstrating safety and efficacy after transplantation. Functional dentin was observed in pulpectomy teeth [104], in the regeneration of 3D dental pulp tissue with blood vessels, and in sensory nerves after traumatic dental injuries [105]. Also, significant clinical improvements with rare adverse effects were observed in the treatment of deep intrabony defects using DP-MSCs [106]. Periodontal tissue regeneration in patients with periodontitis has been observed after treatment with DP-MSCs [107]. Therefore, dental MSCs may be promising for regenerating and repairing dental tissue, although further studies are needed.

It is important to note that the therapeutic potential of DP-MSCs and SHEDs is not limited to dental diseases [97]. A significant number of animal studies have been published using these cells for the treatment of various systemic diseases, such as acute myocardial infarction, systemic lupus erythematosus, Parkinson’s disease, skeletal muscle diseases, liver fibrosis, glaucoma, traumatic spinal cord injury, cerebral ischemic injury, and diabetic neuropathy [108,109,110,111,112,113,114,115,116]. These preclinical data have not been widely translated to human studies as a search of the clinicaltrials.gov database with the keywords “dental pulp stem cells” or “human exfoliated deciduous teeth” and the filters “interventional study” and “cell therapy” resulted in only 5 clinical trials (excluding 15 that have an unknown status or another type of cell or drug associated with it). One of these studies, which was completed in 2015 (NCT01932164), was carried out in Brazil using tissue engineering to repair the alveolar bone defect in patients with cleft lip and palate by mixing DP-MSCs with a collagen and hydroxyapatite biomaterial (Geistlich Bio-Oss^®^). Two other studies conducted in China used DP-MSCs (NCT04130100) and SHEDs (NCT03957655) to assess the clinical efficacy and safety for the treatment of knee osteoarthritis and liver cirrhosis, respectively. To date, no ATMPs using DP-MSCs or SHEDs are commercially available.

### 2.5. Menstrual Blood

Human endometrium is a renewable tissue as it has monthly cyclical events of proliferation, differentiation, and shedding during female reproductive life [117,118]. The extreme periodic turnover and regeneration of endometrium suggests [52] the presence of clonogenic progenitors or stem cells in uterine endometrial tissue. This was confirmed in [52], and some of the cells were found to exhibit MSC-like features [52,119]. Later, a viable MSC population was identified in the menstrual blood fluid by two independent groups [120,121], thus providing a useful new source of MSCs.

Menstrual blood-derived mesenchymal stromal cells (MB-MSCs) are a unique MSC source because they are available monthly throughout the reproductive lifespan; are easily obtained by a safe, noninvasive, and painless procedure; and they can be promptly collected by the donor. Considering that menstruation in women occurs for approximately 30–40 years [122], there are roughly 400 opportunities in each reproductive lifespan [117] to collect menstrual blood and isolate MB-MSCs, thus making these cells an abundant source for clinical use.

The isolation of MB-MSCs can be achieved by directly plating the centrifuged menstrual blood [123,124] or by using density-gradient isolation to recover mononuclear cells [121,125] in a step-by-step procedure similar to that used for BM-MSCs. The isolated MB-MSCs fulfill the BM ISCT criteria for MSCs [126,127,128,129].

MB-MSCs have already been used in several clinical studies of diseases such as multiple sclerosis [130], Duchenne muscular dystrophy, and congestive heart failure [131,132], with no adverse effects. A recent search of the clinicaltrials.gov database using the keywords “menstrual blood mesenchymal cells” or “endometrial regenerative cells” and the filters “interventional study” and “cell therapy” resulted in only five phase I, II, and III clinical trials investigating the use of MB-MSCs in type I diabetes (NCT01496339), cirrhosis (NCT01483248), critical limb ischemia (NCT01558908), COVID-19 (NCT05019287), and poor ovarian response (NTC05703308). These clinical trials are being conducted in China, Iran, and the USA, and they are using either the cells or their secretome. As of October 2023, recruitment was completed for two studies, and the status of the others remains unknown. In 2016, Tan and colleagues conducted a pilot trial using an autologous MB-MSC-based treatment of the uterus of seven patients with severe Asherman’s syndrome (ChiCTR-ONB-15007464), which is characterized by intrauterine adhesions or scar formation that can cause infertility, abnormal placentation, and pregnancy loss. These cells improved the endometrial thickness by up to 7 mm in five women, and 50% of the women treated with MB-MSCs had successful conceptions after frozen embryo transfer. One patient had a spontaneous pregnancy after two local MB-MSC injections [133].

Despite the remarkable therapeutic potential of MB-MSCs, no ATMPs for this category have been registered thus far. However, according to the manufacturing process for endometrial stem cells, there is a need for tests for infectious diseases, cancer, hyperplasia, and endometriosis, etc., to ensure that the isolated MB-MSCs are healthy [134]. Higher passage numbers and donor ages also negatively impact the proliferative capacity of MB-MSCs [128]. Additionally, the isolation method, enrichment protocols, and the day in the menstrual cycle on which the sample is collected must be considered to create a standardized product.

### 2.6. Searching for New Sources: Induced Pluripotent Stem Cell-Derived Mesenchymal Stromal Cells

MSCs derived from human induced pluripotent stem cells (iPSC-MSCs) are a potential source of clinical-grade cell products for regenerative medical applications. iPSCs can proliferate indefinitely and provide extremely large quantities of MSCs for use in cell-based therapy applications. iPSC-MSCs are derived from iPSCs with high proliferative and modulatory profiles, telomerase activity, and paracrine secretion ability. During differentiation, iPSC-MSCs exhibit less senescence and higher survival potential compared to other MSCs under long-term culture and reprogramming protocols [10]. iPSC-MSCs have other clinical advantages such as greater expandability, a reduced heterogeneity due to age- and tissue-related epigenetic changes, and higher regenerative potential [122,123,124].

Many protocols have been established for functional iPSC-MSC generation [135,136,137], and they are compatible with the ISCT criteria for BM-MSCs [47], thus enabling the translational use of iPSC-MSCs in clinical trials to evaluate their potential as cell-based products. A finalized phase I clinical trial conducted in Australia and the United Kingdom (UK) (NCT02923375) showed good results for GvHD using the ATMP Cymerus^TM^, which was approved by the UK regulatory agency. This trial, conducted by Cynata Therapeutics, was a breakthrough as the first clinical trial anywhere involving an iPSC-derived product. Currently a phase II study in GvHD is being conducted (NCT05643638) using CD34-enriched peripheral blood mononuclear-like cells derived from iPSC-MSCs.

Substantial industry interest lies in iPSC-MSC-based ATMPs due to their consistency, scalability, and genetic modification potential. These are important hurdles to overcome in the manufacturing of cell-based therapies to meet industrialized large-scale needs. iPSC-MSCs can be produced on a large-scale and genetically modified to improve their therapeutic potential. The ability to produce a large number of cells in a short period of time with long survival, rapid proliferative capacity, and low batch-to-batch variation is attractive for creating “off-the-shelf” products.

### 2.7. Emerging Technologies and Innovations in MSC Research

The main challenges for the use of MSCs in the clinical scenario are improved survival, retention, homing, immunomodulatory properties, and angiogenesis. To address these issues, researchers are developing new strategies from emerging technologies such as MSC gene editing. The genetic modifications have been widely applied to achieve MSC therapeutical benefits by using viral and non-viral strategies to overexpress the genes related to these processes such as IL-10, HGF, FoxP3, and VEGF [138,139]. An elegantly engineered approach was the use of nanoparticles encapsulated by MSC’s cell membrane to track homing, tumor, or inflammatory tropism [140]. On the other hand, researchers are exploring the paracrine effect of MSCs as their major mechanism of action; in this case, cell-free strategies based on the extracellular vesicles secreted by MSC (MSC-EV) are under investigation. MSC-EV has been shown to have an important role in tissue repair, immune regulation, and anti-fibrotic effects in models of osteoarthritis, spinal cord injury, skin injury, liver fibrosis, kidney fibrosis, and lung fibrosis [141]. Another emerging technology from MSCs’ therapeutical application explores their non-immunogenic properties by using these cells as vehicles to transport chemotherapeutic drugs or cytokines for enhancing in vivo anti-tumoral immunity [138]. They were also used to deliver genes that encode suicide proteins in the context of tumor treatment such as cytosine deaminases, thymidylate kinase from herpex simple or SV40 viruses, and P450 reductase [142,143,144]. These innovations, except for MSC-EVs, are still in the early phases of development and need to reach more robust results to move to clinical settings. These emerging technologies promise to revolutionize the MSC field not only by enhancing MSC biological effects, but also by developing a new role of gene/drug delivery [145].

## 3. ATMP Manufacturing: Challenges Ahead

The number of trials of MSC-based therapies is growing each year, and this field was projected to grow in the market from USD 0.2 billion in 2017 to USD 16.8 in 2024 [146]. Industry-sponsored clinical trials now account for almost 40% of the advanced phase studies, numbering over 100 ongoing projects. The FDA implemented a policy called the Tissue Rapid Inquiry Program (TRIP) from 2017 to March 2021 to encourage regulation of HTC/Ps/ATMPs. However, despite the TRIP initiative, some companies still insist on marketing unapproved ATMP-like products, and this unethical commercialization is evidenced by the amount of consumer complaints submitted to the FDA. In response, the FDA has issued more than 350 letters to clinics and healthcare providers stating that these entities may be offering unapproved regenerative medicine products [147,148].

Even within the MSC-based products approved for commercialization, divergent results have been observed between similar products [53,54]. There is a great need for quality control in the production and cryopreservation of ATMPs on an industrial scale. It is urgent to regulate the production of MSCs as a defined product, with its identity, potency, and stability attested by validated analytical methods. Furthermore, the route of administration must also be taken into consideration to maximize the desired MSC biological activity. In other words, the standardization of protocols used to generate and apply MSC products is necessary to deliver optimal patient care in this rapidly emerging field of regenerative medicine, and this will be discussed in the following section.

### 3.1. Establishment of Identity

The clinical effect of MSCs can be influenced by several factors, including the donor, the tissue source, cell quantity, and/or bioactivity and the manufacturing process, thus making the definition of MSC-product identity mandatory. This requires steps that begin in basic research, pass through quality control, and culminate in delivery of the product to the market.

Donors are a special source of intrinsic variation for ATMPs due to genetics, age, sex, and environmental factors (e.g., stress, body mass index, etc.), all of which may influence the performance of the obtained cells. Choice of tissue source for cell isolation also affects the variation of ATMPs since tissues are living entities responding to various environmental stimuli that influence their therapeutic actions. An example is MB-MSCs, which require common tests that are applied to all MSC sources for clinical and laboratory screening (infectious diseases, cancer, and hyperplasia) but also include endometriosis assessment and information about the day in the menstrual cycle at sample collection [134]. As noted above, higher passage numbers and donor ages also negatively impact MB-MSC proliferative capacity [128]. The senescence of MSCs in culture is one of the characteristics influenced by donor characteristics and cell sources [149,150].

The immunophenotypes of MSCs obtained from different sources show slight differences, as described above. Scientific societies need to define cell identity to standardize this process and create a universal reference for regulatory agencies. Following the example of ISCTs for BM-MSCs, some movement in this direction has begun and should be widely supported. In 2008, the First International Workshop on Placenta-Derived Stem Cells suggested minimal criteria for defining the MSCs derived from human placenta (P-MSCs) and umbilical cord (UC-MSCs), which were based on standards already established for BM-MSCs by the ISCT, with an additional criterion that the cells must be of fetal origin [57]. In 2013, the International Federation for Adipose Therapeutics and Science proposed minimum criteria for identifying adipose stem cells (AT-MSC) based on standards already established for bone marrow by the ISCT [151].

### 3.2. The Manufacturing Process

The isolation method, the enrichment protocols, and the cell culture expansion protocols must be defined to create a standardized product. Alterations in enzymatic digestion, like time and enzyme type, can impact cell viability and yield, as well as financial costs [152,153,154,155,156]. The development of a defined medium and recombinant supplements were a milestone in the translational application of these cells. These standardized media result in less batch-to-batch variability, and the use of reagents of non-animal origin avoids the risk of pathogen transmission and xeno-associated immune reactions [157].

Control of the cell cycle is necessary to ensure correct cell development and function. In vitro studies demonstrate that cell passage and density influence the proliferation rate; for example, younger cells at lower density proliferate faster [158]. Another important factor that decreases cell proliferation rate is time in culture, which affects telomerase activity and leads to morphological and molecular changes in the cell. Understanding the cell cycle is essential to determine the passage for cell infusion into the patient, thereby guaranteeing the safety of using a chromosomally stable cell population [149,159].

To ensure the reproducibility of the ATMP manufacturing process, both the FDA and EMA highly recommend the establishment of master cell banks and working cell banks. These agencies advocate that cell-based ATMPs must be prepared by expanding the cells obtained from master cell banks [160,161]. These banks would have control in terms of the number of passages in culture; the number of cells per vial, identity, viability, and morphology; and they would ensure the cells are free of viruses, bacteria, endotoxin, mycoplasma, and fungi. Also, stability tests would guarantee MSC integrity and biological activity under storage conditions, thus providing greater security about the origin of the cell.

The use of good manufacturing practice (GMP) in the cell development process is mandatory for clinical use. Standardized and further optimized procedures with stringent control must be applied, such as the use of aseptic methods, xeno-free culture media, defined supplements, and subculturing conditions. Moreover, cryopreservation processes, when used, must also be included in GMP standards. It has already been demonstrated that freezing/thawing has a significant negative impact on the therapeutic effect of MSCs compared to fresh cells [54]. In summary, GMP protocols for cryopreservation require optimization and quality review to ensure that the MSC-based product is of a high functional quality. GMP cryopreservation standards are a major step toward optimizing the entire production process for a stable and homogeneous product.

In addition, the definition of the best method to determine critical cell properties could be standardized by societies and regulatory agencies, which would represent a benefit to the field. The choice of some analytical methodologies could facilitate comparative results between different labs and even minimize batch differences. For instance, the population doubling time assay could be more adequate and reproducible to evaluate cell proliferation/senescence than counting passages, i.e., how many enzymatic digestions the culture cells were submitted to. The same reasoning could be applied to the vast majority of assays used to produce MSCs. The main challenges and possible solutions regarding the use of MSCs as an ATPM are summarized in Table 2.

### 3.3. Challenges in Selecting a Potency Assay

The potency tests for MSC-based products are complex. These assays must demonstrate the biological activity of each particular intended application correlated to the expected clinical effect. The potency test also must detect functionally important differences between product lots [163,164,165].

In the USA, the definition of potency is “the specific ability or capacity of the product, as indicated by appropriate laboratory tests or by adequately controlled clinical data obtained through the administration of the product in the manner intended, to effect a given result” (21 CFR 600.3(s)). In the context of MSC-based products, the potency assay would determine whether the product was produced under controlled manufacturing processes ensuring MSC identity, purity, stability, and biological activity. According to the FDA, these tests should be performed in vitro, in vivo, or both, and they should ensure that all MSC products are released to meet the acceptance criteria of defined specifications [160].

Although extreme effort has been made to understand the mechanisms of MSC function during tissue repair, these processes remain to be fully understood [76,166]. The main biological activity expected from an MSC-based therapy would be dependent on the microenvironment into which the cells are administered. For example, the Mechanism of Action studies demonstrated that the multipotentiality of MSCs may not be essential for the improvement of symptoms of osteogenesis imperfecta [167,168]. Moreover, the transient presence of cells transplanted into the eye suggests that paracrine action is essential for the therapeutic effect observed in retinal degeneration [169,170]. Several studies have demonstrated that improvements in animal models of degenerative diseases, such as acute renal failure, liver cirrhosis, myocardial infarction, spinal cord injury, or the multifunctional complications associated with diabetes, are related to the ability of MSCs to produce and secrete trophic factors (i.e., HGF, IGF-1, VEGF, VEGF-a induced by zeolitic imidazolate framework 8, EPO, and GDNF [50,171]). This plethora of biological activities in distinct organs and disease states imposes a huge challenge when choosing a potency assay to validate the biological activity intended in the context of a specific disease, and they must be defined individually.

### 3.4. Route of Administration and Impact of Delivery Mechanisms

Preclinical and clinical reports describe different routes of administration of MSCs. Systemic delivery through intravenous injection covers most target diseases, with promising outcomes in neurological injuries [172]. However, different injection routes could affect safety and efficacy, which are still under discussion [173]. An example of this is local injections yielding potentially increased beneficial effects, as observed in the newly approved AT-MSCbased ATMP Darvadstrocel, which is given as an enterocutaneous injection for fistular disease. Nevertheless, other data demonstrate that the local injection of BM-MSCs into an infarcted heart could worsen the disease [174]. No conclusive in vivo evidence exists to show that direct tissue tropism is required for the beneficial effects of MSCs in improving tissue repair or inflammatory syndromes. Furthermore, there is a vast body of literature that indicates that the expression of functional adhesion molecules, chemokine receptors, and metalloproteinases, which are indispensable for the trafficking of MSCs from the vessel to the target tissue, are deficient, thus impairing MSC homing/migration [175]. Further investigations are needed to clarify these questions.

### 3.5. Biosafety Profile of MSC

Regardless of the treated disease, tissue source, autologous or allogeneic treatment, and route of administration, the clinical trials showed that the MSC therapy is safe and unrelated to genetic abnormality induction, tumor formation, or expressive host immune response. The meta-analysis studies were performed by combining the data from clinical trials, which was achieved by mainly exploring the safety of MSCs and how their heterogeneity influences clinical outcomes including BM-MSC, UC-MSC, and AT-MSCs as cell products in various diseases [176,177,178]. The results from these studies suggest there may be transient fevers in patients who receive MSC. Nevertheless, the therapy was not related to severe adverse events such as vascular disorders, urticaria/dermatitis, central nervous system disorders, diarrhea, infection, or death, even when high doses were administered [179,180]. In addition, Wang et al. demonstrated that the myocardial-infarcted patients tended to present lower rates of arrhythmia when treated with MSCs [176]. These analyses confirm the safety of MSC therapy; for a more detailed analysis, we suggest Wang et al. 2021 [176].

## 4. The COVID-19 Experience

Since the beginning of the coronavirus disease (COVID-19) outbreak until its pandemic status, several MSC-based clinical trials were registered as an alternative treatment for severe COVID-19 respiratory disease. Subsequently, there has been a rapid global increase in COVID-19-related MSC trials, thus corroborating the promise of MSC treatment for inflammatory and immune diseases [74]. This explosion of studies is justified by the well-known immunomodulatory properties of MSCs and the fact that the lung retains systemically injected cells (albeit for a short period) [181,182]. There is a great expectation of success based on the successful data of MSC therapy on immune/inflammatory conditions [74].

The use of MSCs in COVID-19 relies on their paracrine profile [183]. This disease progresses in phases where, initially, an enormous inflammatory response is stimulated. A few days later, a cytokine storm occurs, which is where MSCs can make a difference as inflammatory regulators. It has already been demonstrated that the inflammatory milieu enhances the immunosuppressive function of MSCs by reinforcing the host’s own regulatory/immunosuppressive immune subsets [184]. This scenario could be compared to the successful use of MSCs on GvHD inflammation [185].

One of the first studies to post results (NCT04252118) was a phase I Chinese study that enrolled 18 patients with COVID-19. The treatment group received an intravenous infusion of UC-MSCs and standard COVID-19 care regimens. The results demonstrated that cell therapy in patients with moderate and severe COVID-19 is safe and well tolerated, thus reinforcing the necessity for randomized, controlled, and double-blinded phase II and III trials with long-term follow-ups to evaluate the therapeutic use of UC-MSCs in COVID-19 [186]. Specifically, the study found that patients who received the cell therapy with UC-MSCs required less mechanical support ventilation during their hospitalization compared to those in the control group. Additionally, the cell therapy reduced clinical symptoms such as coughing and shortness of breath. Notably, patients who underwent the cell therapy treatment showed reduced IL-6 serum levels, a faster recovery of the lung parenchyma, and better control of lung lesions when compared to the control group [186]. To date, the clinicaltrials.gov website has 96 trials registered worldwide using MSCs. Most use extraembryonic P-MSCs or UC-MSCs, but AT-MSCs, BM-MSCs, DP-MSCs, MB-MSCs, and iPSC-MSCs are also under investigation. MSCs obtained from different sources have shown consistent outcomes in clinical trials. In general, studies have shown that cell therapy with MSC-derived products reduces the levels of C-reactive protein and stabilizes the levels of liver enzymes and circulating immune cells. In addition, a remarkable and common outcome after treatment is the restoration of oxygenation, an increase in PaO2/FiO2, and a downregulation of the cytokine storm. Fortunately, these results contribute to increased survival rates and confirm that MSCs are important candidates for the treatment of diseases, such as COVID-19 (NCT04573270, NCT04398303, NCT04452097, and NCT04537351).

As a product derived from iPSCs, there is a clear concern regarding the presence of residual pluripotent stem cells in the final product. However, it is important to keep in mind that specific tests must be conducted to mitigate the risk of tumorigenesis, which is a major concern regarding the use of iPSC-derived products. According to the FDA, comprehensive preclinical testing conducted in animals to assess tumorigenicity and risk assessment should be performed to ensure safety and to confirm that there is no residual, undifferentiated iPSCs that are persistent in the final product. The FDA advises that the tumorigenicity model should be able to show cell survival for a sufficient time period. In line with the FDA’s 2013 guidance document [187], important aspects should be considered such as (i) the differentiation status profile (ranging from undifferentiated/embryonic to terminally differentiated/specialized cell type); (ii) the extent of cell manipulation employed during the iPSC-derived product manufacture (including details about the growth kinetic profile); (iii) the expressed transgene of genetically modified cells; (iv) the potential to induce or enhance tumor formation from existing subclinical host malignant cells; and (v) the target patient population. Furthermore, optimizing the cocktail of reprogramming factors using strategies based on chemical-inducing reprogramming, eliminating aberrant cells via a drug-inducible elimination system, and increasing the purity of differentiated cells derived from iPSCs samples are efficient strategies for reducing tumorigenicity risk [188].

## 5. Conclusions

MSC-based therapy in clinical practice is already a reality. However, MSC-based therapy still faces barriers as the field awaits effective regulation. The identity and the mechanism of action of MSCs are still areas of intense investigation and debate. Numerous morphological, phenotypic, and molecular parameters about the identity and heterogeneity of MSCs have been described in the literature, but this topic is still far from reaching a consensus. The choice of an effective cell potency assay is also challenging since MSCs are used to treat several diseases where different mechanisms of action are expected, and it is highly unlikely that a single assay will be suitable for all clinical applications.

For MSC-based therapy to advance in the regenerative medicine field and beyond, close interactions and coordinated efforts among the four main drivers of the technology are imperative: basic researchers, clinicians, sponsors/industry, and regulatory agencies need to lead this initiative collaboratively. 

## Figures and Tables

**Table 1:** MSC-based therapies classified as ATMPs.

Name	Cellular Type	Sponsor	Clinical Indication	Agency and Year of Approval
Prochymal (Remestemcel-L/Ryoncil)	BM-MSC	Mesoblast	GvHD	Health Canada, 2012 and US FDA, 2015
Cellgram-AMI	BM-MSC	FCB Pharmicell	AMI	South Korea MFDS, 2011
Neuronata-R	BM-MSC	Corestem	ALS (Lou Gehrig’s Disease)	South Korea MFDS, 2014
Stempeucel	BM-MSC	Stempeutics Research	CLI	India DCGI, 2016
Temcell HS	BM-MSC	JCR Pharmaceuticals	GvHD	Japan PMDA, 2015
MesestroCell	BM-MSC	Cell Tech Pharmed	Knee osteoarthritis	Iran FDA, 2018
Alofisel (darvadstrocel)	AT-MSC	Tigenix/Takeda	CD Perianal Fistula	Europe EMA, 2018
Cupistem	AT-MSC	Anterogen	CD Perianal Fistula	South Korea MFDS, 2012
Cymerus^TM^	iPSC-MSC	Cynata	GvHD	UK MHRA, 2016
Cartistem	UC-MSC	Medipost	Knee osteoarthritis	South Korea MFDS, 2012
Stemirac	BM-MSC	Nipro Corp	Spinal cord injury	Japan PMDA, 2018

GvHD, graft-versus-host disease; AMI, acute myocardial infarction; ALS, amyotrophic lateral sclerosis; CLI, critical limb ischemia; and CD, Crohn’s disease. Adapted from Ramezankhani et al. 2020 [34].

**Table 2:** Challenges and possible solutions for the use of MSCs as an ATMP.

Cell Source	Challenges	Possible Solutions	References
Bone marrow	Inter-donor and product variabilities Senescence, loss of proliferation, and phenotypical changes during expansion	Follow regulatory agencies and ISCT guidelines Establishment of standard protocol and characterizations Robust donor selection Continuous process verification Risk assessment checkpoints during manufacturing process	[34,49,53,54,55,56,162]
Placental and Umbilical cord	Tissue donation and procurement Several expansion steps needed to produce a sufficient quantity for clinical use Inter-donor and product variabilities Manufacturing standardization	Follow regulatory agencies and ISCT guidelines Produce unlimited cell banks Establishment of standard protocol and characterizations Robust donor selection	[64,75,76,77,78,162]
Adipose tissue	Inter-donor and product variabilities Isolation protocol Manufacturing standardization	Follow regulatory agencies and ISCT guidelines Establishment of standard protocol and characterizations Robust donor selection Risk assessment checkpoints during manufacturing process	[82,83,84,85,86,87,162]
Dental pulp	Few clinical trials Manufacturing standardization No ATMP commercially available	Follow regulatory agencies and ISCT guidelines Translational and clinical studies to consolidate results	[87,88,98,162]
Menstrual blood	No ATMP commercially available Cell obtention from healthy patients Low proliferative capacity after higher passages Inter-donor variabilities Manufacturing standardization	Follow regulatory agencies and ISCT guidelines Robust donor selection Implement standard protocol establishment by taking into account the day of the menstrual cycle to perform cell obtention Continuous process verification Follow regulatory agencies guidelines and recommendations	[127,128,129,130,135,162]
iPSC-MSC	Risk of residual iPSC in final product Manufacturing standardization	Robust preclinical tests and tumorigenicity risk assessment Continuous process verification Establishment of standard protocol and characterizations	[122,123,124,136,137,146,162]

## Data Availability

Not applicable.
